# Overexpression long non-coding RNA *LINC00673* is associated with poor prognosis and promotes invasion and metastasis in tongue squamous cell carcinoma

**DOI:** 10.18632/oncotarget.14200

**Published:** 2016-12-25

**Authors:** Jianjun Yu, Yan Liu, Zhaojian Gong, Shanshan Zhang, Can Guo, Xiayu Li, Yanyan Tang, Liting Yang, Yi He, Fang Wei, Yumin Wang, Qianjin Liao, Wenling Zhang, Xiaoling Li, Yong Li, Guiyuan Li, Wei Xiong, Zhaoyang Zeng

**Affiliations:** ^1^ Department of Head and Neck Surgery, Hunan Cancer Hospital and The Affiliated Cancer Hospital of Xiangya School of Medicine, Central South University, Changsha, Hunan, China; ^2^ The Key Laboratory of Carcinogenesis of the Chinese Ministry of Health, Cancer Research Institute, Central South University, Changsha, Hunan, China; ^3^ The Key Laboratory of Carcinogenesis and Cancer Invasion of the Chinese Ministry of Education, Xiangya Hospital, Central South University, Changsha, Hunan, China; ^4^ Department of Stomatolog, The Second Xiangya Hospital, Central South University, Changsha, Hunan, China; ^5^ Hunan Key Laboratory of Nonresolving Inflammation and Cancer, Disease Genome Research Center, The Third Xiangya Hospital, Central South University, Changsha, Hunan, China; ^6^ Department of Cancer Biology, Lerner Research Institute, Cleveland Clinic, Cleveland, Ohio, USA

**Keywords:** long non-coding RNA (lncRNA), long intergenic non-coding RNA 673 (LINC00673), tongue squamous cell carcinoma (TSCC), prognosis, metastasis

## Abstract

Long non-coding RNAs (lncRNAs) associated with the tumorigenesis of human cancers. However, the relevance of lncRNAs in tongue squamous cell carcinoma (TSCC) is still unclear. To discover novel TSCC-related lncRNAs, we analyzed the lncRNA expression patterns in two sets of TSCC gene expression profile data, and found that long intergenic non-coding RNA 673 (*LINC00673*) was significantly upregulated in TSCC samples. Then we examined *LINC00673* expression in 202 TSCC tissue specimens, *LINC00673* is highly expressed in a significant proportion of human TSCC biopsies and correlates with poor prognosis. Knockdown *LINC00673* significantly inhibited the cell invasion and migration capability in TSCC cells. Our findings suggest that *LINC00673* may play an essential role in TSCC progression and might serve as a potential biomarker for early detection and prognosis prediction of TSCC.

## INTRODUCTION

Head and neck cancer is the sixth most common malignant tumors all around the world, accounting for approximately 6% of all cancers mortality [1–3]. Epithelial tumors arising in the oral cavity is the most high-frequently occurred cancer in head and neck region, and tongue squamous cell carcinoma (TSCC) is the most common histological malignancy of in this region [4]. TSCC comprises 25% to 40% of oral cancers and is the main cause of this type cancers mortality [5–7]. In spite of improvements in diagnostic tools and treatment prescription such as operation, chemotherapy and radiotherapy over the past few decades, TSCC cells extraordinary local invasion and lymphnode metastasis still are enormous challenges of the disease course of treatment, and it still has a high risk of developing secondary or recurrent tumors in the surrounding area and overall survival rates of advanced (stage III-IV) of it is less than 65% [8–10].

Long non-coding RNA (lncRNA) is a newly-identified class of RNAs that are more than 200 nucleotides in length and without protein-encoding ability. Accumulating evidence has shown that they play crucial roles in many physiological processes, such as carcinogenesis, by modulating gene expression at the epigenetic, transcriptional and posttranscriptional levels [11–15]. Furthermore, recent studies have identified multiple functional effects of lncRNAs involving in various cellular processes, such as tumor initiation, progression and metastasis, and the dysregulation of lncRNAs are a primary feature of some human cancers [16–19]. At the same time, a great quantity identified results revealed that lncRNAs play an important role in pathogenesis of head and neck cancers [20–22]. However, the TSCC associated lncRNAs and their influence on TSCC cellular progress need to be elucidated further.

In this study, we aim to profile expression patterns and dysregulation of lncRNAs in TSCC by analyzing two previously published TSCC data sets (GSE9844 [23] and GSE30784 [24]), based on Affymetrix gene expression microarray platform. One lncRNA, long intergenic non-coding RNA 673 (*LINC00673*) was significantly up-regulated in TSCC tissues than that in corresponding non-tumor tissues. Then, we examined its expression in fresh TSCC biopsies and paraffin-embedded tissues by quantitative real time polymerase chain reaction (qRT-PCR) and in situ hybridization, respectively. Furthermore, we performed in vitro knockdown experiments inhibiting *LINC00673* expression in TSCC cells to assess the changes in tumor cell behavior when *LINC00673* expression is lost.

This study represents a significant step forward in understanding the importance of lncRNAs in TSCC, and provided a novel insight concerning the role of *LINC00673 in* the progression of TSCC. Future studies based on these findings may lead to discover novel TSCC biomarkers or targeted therapies.

## RESULTS

### The expression profile of lncRNAs in TSCC tissues

**Figure 1:**
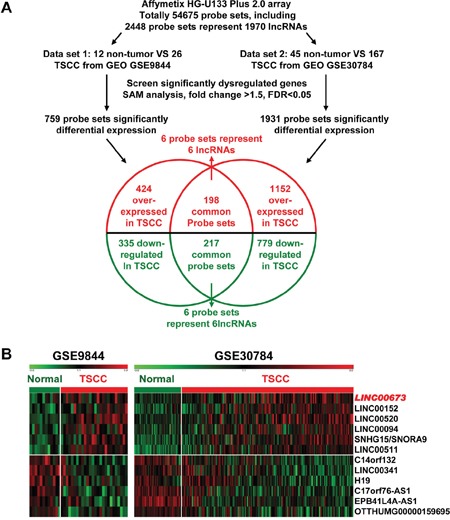
Dysregulated lncRNAs expression analysis using two independent TSCC cohorts and cDNA microarray analysis **A**. Schematic overview of the workflow used to identify dysregulated lncRNAs in two TSCC microarray data cohorts (GSE9844 and GSE30784). **B**. Heatmap of 12 dysregulated lncRNAs mined from the GEO data set.

In an attempt to identify novel long non-coding RNA in TSCC, two GEO datasets GSE9844 and GSE30784 were used to explore the lncRNA's differential expression between TSCC tissues and normal lingual mucous membrane (Figure 1A). Through aggregation of differentially expressed lncRNAs signatures from these two GEO datasets, 12 overlapping probesets, revealing 6 upregulated and 6 downregulated lncRNAs (Figure 1B). Most were unknown and not well identified, such as *LINC00673*, *LINC00152*, *LINC00520*, *LIN00094*, *LINC00511*, *EPB41L4A-AS1*, and *LINC00341*. *H19* [25–31], a famous oncogenic lncRNA in many cancers, also listed and overexpressed in TSCC samples among these overlapping probe sets. To investigate the role of lncRNAs in TSCC tumorigenesis, we focused on *LINC00673*, for which the expression was most significantly different and had never been reported in human cancer.

### *LINC00673* is high expressed in TSCC

**Figure 2:**
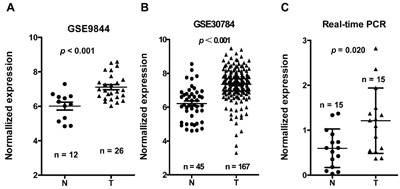
*LINC00673* expression was upregulated in three independent cohorts of TSCC biopsies *LINC00673* expression, as measured by Affymetrix microarray, was upregulated in TSCC biopsies when compared with non-tumor lingual mucous membrane tissues in GSE30784 **A**. and GSE9844 **B. C**. *LINC00673* was upregulated in TSCC through measured in 15 paired TSCC tissues and adjacent non-tumor tissues using quantitative PCR (qRT-PCR). T, tumor; N, non-tumor tissues.

*LINC00673* expression was one of the most significantly upregulated in the TSCC tissues compared to non-tumor tissues according to the GSE9844 and GS30784 datasets (Figure 2A & 2B, *p* < 0.001 in both datasets). To assess the role of *LINC00673 in* TSCC progression, the expression levels of *LINC00673 in* 15 paired TSCC tissues and adjacent non-tumor tissues were tested by qRT-PCR. Results showed that the transcript levels of *LINC00673 in* TSCC tissues were significantly up-regulated compared to that in adjacent normal tissues (*p* = 0.020, Figure 2C), which was consistent with the GEO datasets.

### Correlations between aberrant expression of *LINC00673* and TSCC clinical pathological parameters

**Figure 3:**
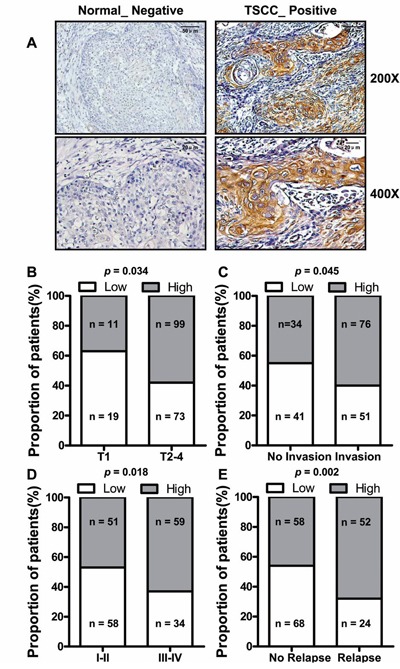
The relationship between *LINC00673* and pathological clinical feature **A**. *LINC00673* expression was measured by *in situ* hybridization in paraffin embedded TSCC biopsies (T, n = 202). Representative cases of non-tumor lingual mucous membrane (N) and TSCC biopsies are shown. Proportion of TSCC patients with *LINC00673* expression shown that upregulated *LINC00673* was positively correlated with tumor size **B**., tongue muscle invasion **C**., TNM stage **D**. and relapse **E**.

*In situ* hybridization was used to evaluate correlation of *LINC00673* expression level such as with patients’ clinicopathological parameters in TSCC, such as tumor size (T stage), lymph-vascular invasion (N stage), invasion muscles of tongue, pathological stage, relapse and so on. Representative images of *LINC00673* signals are shown in Figure 3A. High *LINC00673* expression was positively correlated with Tumor size (*p =* 0.034, Figure 3B), invasion muscles of tongue (*p* = 0.045, Figure 3C), higher TNM stage (*p* = 0.018, Figure 3D), and relapse (*p =* 0.002, Figure 3E). However, other clinical parameters, such as gender, age, smoking and chewing areca, were found not to be significantly correlated with *LINC00673* expression in this study. The detailed results of clinical parameters and expressions were shown in Supplemental Table 1.

### High expression of *LINC00673* predicts poor prognosis in TSCC

**Figure 4:**
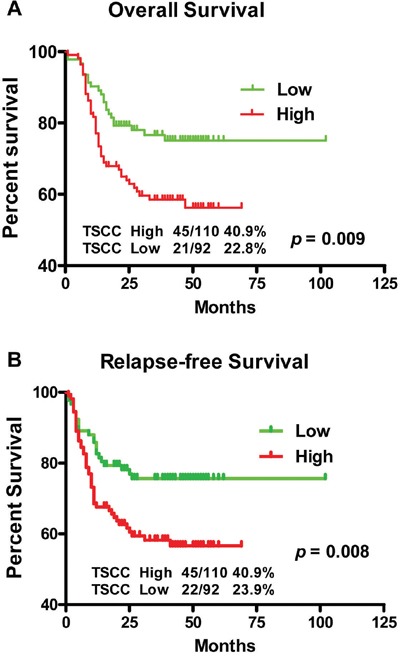
Kaplan-Meier survival curves in TSCC patients according to *LINC00673* expression levels The highly expressed *LINC00673* was correlated with shorter overall survival **A**. or relapse free survival **B**. of TSCC patients.

Additionally exploring of the correlation between *LINC00673* expression and clinical outcomes demonstrated that, the median overall survival (OS) time was 39.5 and 29 months in TSCC patients with low and high expression of *LINC00673*, respectively (Figure 4A, *p* = 0.009), while the median relapse-free survival (RFS) time of TSCC patients with low and high expression of *LINC00673* was 39 and 25 months, respectively (Figure 4B, *p* = 0.008). High expression of *LINC00673* was associated with poor overall survival and poor relapse-free survival, and could be regarded as an independent predictor for overall survival in TSCC

### Knockdown *LINC00673* inhibits TSCC cell migration and invasion

**Figure 5:**
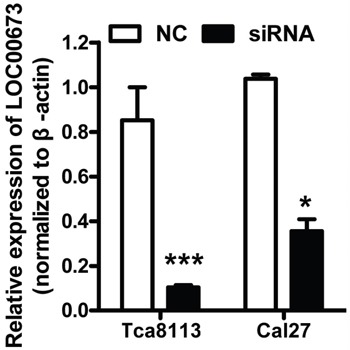
siRNAs successfully knockdowned *LINC00673* expression Tca8113 and Cal27 cells were transfected with siRNAs targeting *LINC00673*. *LINC00673* expression was significantly decreased when measured by qRT-PCR.

**Figure 6:**
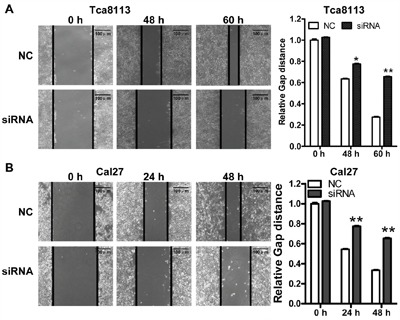
Knockdown *LINC00673* suppressed TSCC tumor cell migration Wound healing assays demonstrated that the migratory potential of Tca8113 **A**. and Cal27 **B**. cells were significantly reduced by siRNAs specifically targeting *LINC00673* (siRNAs) when compared with scrambled control siRNA (NC) treated cells.

**Figure 7:**
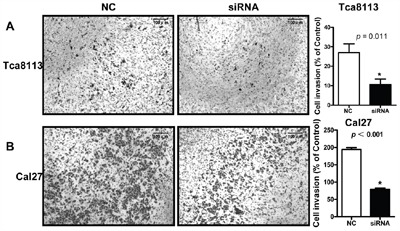
Knockdown *LINC00673* suppressed TSCC tumor cell invasion Transwell assay results showed that the invasion potential of *LINC00673*-silenced Tca8113 **A**. and Cal27 **B**. cells (siRNAs) were significantly reduced when compared with the control group (NC).

To explored the function of *LINC00673 in* TSCC, we knockdown *LINC00673* expression by using two *LINC00673* targeting short interfering RNAs (siRNAs) in TSCC cell line, Tca8113 and Cal27. qRT-PCR results indicated that *LINC00673* expression were significantly inhibited in both Tca8113 and Cal27 cells after siRNAs transfection (Figure 5). Wound healing assays demonstrated that the migratory potential of *LINC00673*-silenced cells were significantly reduced when compared with scrambled control siRNA treated (Figure 6). The transwell assay results showed that knockdown of *LINC00673* inhibited Tca8113 and Cal27 cells invasion and compared to the control group (Figure 7).

## DISCUSSION

Recent reports indicate that lncRNAs play a critical role in a wide range of tumor progression such as differentiation, proliferation, and metastasis [32–34]. The pathogenesis and carcinogenesis of TSCC are multi-step and heterogeneous processes, and involve different genetic and epigenetic changes [35]. A number of lncRNAs have been discovered to play a vital role in carcinogenesis and progression of TSCC [36], including, *HOTTIP* [37], *MALAT1* [38], *TUC338* [39], *UCA1* [40], etc. In the current study, we combined two previously published GEO dataset to identify differentially expressed lncRNAs in TSCC. We found 12 differentially expressed lncRNAs in TSCC tissues when compared with the noncancerous tissue samples. Among these 12 lncRNAs, *LINC00152* had been reported that differentially expressed and participating in tumorigenesis in many tumors, was also included and upregulated in TSCC samples compared with the adjacent non-tumor tissues [41–43]; H19, another famous lncRNA, was also listed [25–31]. While other ten lncRNAs have not been well characterized. These dysregulated lncRNAs might have function in the carcinogensis of TSCC and more functional study is needed to identify valuable and effective lncRNAs-based diagnosis, prognosis and therapeutic biomarkers in TSCC.

In this study, we chose and focused on *LINC00673* since it was significantly high express among lncRNAs dysregulated in TSCC but never been reported in TSCC previously. We performed qRT-PCR and *in situ* hybridization to verify our results using existing data in the GEO database. We found *LINC00673* was upregulated in TSCC and it's upregulation was associated with tumor progression, such as tumor size, invasion of muscles, clinical stages, recurrence and poor survival. After siRNA-mediated silencing of *LINC00673*, we found that could significantly inhibit TSCC cancer cell invasive and migratory abilities. In recent findings, some lncRNAs, such as actin filament associated protein 1 antisense RNA1 (*AFAP1-AS1*) [44, 45], HOX transcript antisense RNA (*HOTAIR*) [46], growth-arrest-specific transcript 5 (*GAS5*) [47], have been reported to regulate cancer metastasis process by multiple ways. The potential role of *LINC00673* abnormalities in TSCC is worth for further investigated.

In conclusion, our findings indicate that *LINC00673* might be involved in TSCC development, which may play a vital role in the tumorigenesis of TSCC, and may serve a useful biomarker for the prediction of TSCC prognosis and as potential target for therapy in the future.

## MATERIALS AND METHODS

### Data mining and analysis

Two independent cohorts of primary TSCC gene expression profiling (GEP) data based on the Affymetrix Human Genome U133 Plus 2.0 platform, GSE9844 [23] and GSE30784 [24], and their correlated clinic data were obtained from the Gene Expression Omnibus (GEO) database (http://www.ncbi.nlm.nih.gov/geo/). GSE9844 contain 26 cancer tissues and 12 normal adjacent tissues. GSE30784 has 167 cancer tissues and 45 normal adjacent tissues. Significant Analysis of Microarray (SAM) [48–51] software was used to analyze the different expression of lncRNAs between normal lingual mucous membrane and TSCC tissue samples in these two published TSCC datasets. The cut-off value for differentially expressed lncRNA was set at ≥ 1.5-fold change and the false discovery ratio (FDR) was < 0.05. The data analysis procedures are shown in Figure 1A.

### Clinical samples

Two sets of clinical samples were collected for this study: Set 1 for qRT-PCR, containing 15 paired primary TSCC tissues and adjacent non-tumor lingual mucous membrane biopsies, collected from TSCC patients when they underwent surgical resection. None of the patients had received radiotherapy or chemotherapy prior to surgery. Set 2 for *in situ* hybridization, paraffin-embedded TSCC tissue samples from 202 cases of patients who underwent surgical operation from January 2009 to December 2013 at the Affiliated Cancer Hospital of Central South University. Clinical data were reported in Supplemental Table 1. This study was approved by the Ethical Committee at the Affiliated Cancer Hospital of Central South University and every patient provided the written informed consent.

### RNA isolation and qRT-PCR

Total RNAs were extracted using TRIzol reagent (Invitrogen, Carlbad, CA, USA). One μg of total RNA from the samples was reverse transcribed using a Reverse Transcription Kit (Biorad, Hercules, CA, USA). Real-time PCR was performed using SYBRGreen (Biorad) in the CFX96 Real-Time PCR Detection System (Bio-Rad) [52–54]. The primers used were *LINC00673*: 5′-TTCTCC TGTAACGTGTGGCC-3′ and 5′-CTGGTGGGAATGTGGATCA GT-3′; *ACTB* (β-actin): 5′-TCACCAACTGGGACGACATG-3′ and 5′-GTCACCGGAGTC CATCACGAT -3′. *ACTB* was used as the reference and normalization control. *LINC00673* expression was normalized to the respective *ACTB* expression level. Relative expression was calculated using the equation: ΔCt = Ct (target gene) – Ct (*ACTB*), fold expression = 2^–(ΔCt(tumor) – ΔCt(normal))^.

### *In situ* hybridization

*In situ* hybridization was performed to detect *LINC00673* expression as previously described [55–57]. Three probes from different *LINC00673* regions (5′-GAAAAACCTCTTG CACCACCTTAGTCTCCAAAGA-3′, 5′-CTTTCCTGTTCTTTCTCCTACCCTTCCTGAC TAG -3′, and 5′-CATGAAGTAATAATAAAGGTTCCGCTTATCAACC-3′) were synthesized and labeled with DIG-dUTP at the 3′ end (Invitrogen, Shanghai, China). Three *GAPDH* probes used as positive controls were 5′-CCACTTTACCAGAGTTAAAAGCAGCCCTGG-3′, 5′-CAGTAGAGGCAGGGATGATGTTCTGGAGAG-3′, and 5′-GTCAGAGGAGACC ACCTGGTGCTCAGTGTA-3′. A semi-quantitative scoring criterion for *in situ* hybridization was used in which both the staining intensity and the number of positive cells were recorded. The scoring was graded as 0 (negative), 1 (< 10% positive), 2 (10% - 50% positive), or 3 (> 50% positive) in accordance with the staining proportion and intensity. The final scores were regarded as low expression (0-1) and high expression (2-3). All sections were scored in randomly selected five representative fields of vision at medium magnification by two pathologists who were blinded to the clinicopathological features and the clinical data.

### Cell line and gene silencing

The TSCC cell lines Tca8113 and Cal27 were maintained in an atmosphere of 5 % CO_2_ at 37°C and cultured in RPMI 1640 medium supplemented with 1 % antibiotics (100U/ml penicillin and100 μg/ml streptomycin sulfates) and 10 % fetal bovine serum (FBS, GIBCO).

For gene knockdown, cells were seed in six-well plates to confluency and transfected siRNAs by using Lipofectamine RNAiMAX Reagent (Invitrogen) in OptiMEM medium (Invitrogen) [58–61]. Two sequences of *LINC00673* targeting siRNAs were: 5′-CCAGTTGTC CTTGACTGCATGGTTT-3′; and 5′-AGGGAACCACAGGATTCCATGTGAT-3′. Sequences of non-target scramble controls were provided by Invitrogen.

### Cell migration and invasion assay

Wound healing assay was used to examine TSCC cells invasion capacity. The cells were seeded in six-well culture plates and grown to 90 % confluence. Vertical wounds in the cell monolayer were created by a 10 ul tip and washed three times with PBS to remove cell debris. Wound width was measured by microscopy at the designated time periods [62–65].

Transwell assay was used to assess tumor cell migration capacity. A total of 1×10^5^ cells in 100 μl of serum-free medium were added to the top of transwell Cell Culture chambers (8 μm pore size, BD Biosciences, New Jersey, USA), and 600 μl of 10% FBS containing medium was added to the lower chamber. Cells were incubated for 36 h at 37°C, and then, the migrated cells were fixed with methanol, stained with 0.5% crystal violet. Cells on the upper surface were wiped by a cotton bud. Numbers of invasive cells were counted from six randomly selected 200× fields in under a microscope and shown as the average per field [66–68].

### Statistical analysis

All experiments were independently repeated at least triplicate. All the Statistical analysis was carried out by using SPSS software, version 19.0 (SPSS, Chicago, IL, USA) and presented with Graph-pad prism software. Differences between two independent groups were evaluated by Student's t-tests and differences for multiple comparisons were evaluate by one way ANOVA. Overall survival (OS) or relapse-free survival (RFS) were calculated using the Kaplan-Meier method, and the results of the analysis were considered significant in a log-rank test if *p* < 0.05. All data are represented as means ± standard deviation. A two-tailed p value of 0.05 or less was considered statistically significant.

## SUPPLEMENTARY TABLES





## Abbreviations

lncRNA: long non-coding RNA
TSCC: tongue squamous cell carcinoma
*LINC00673*: long intergenic non-coding RNA 673
qRT-PCR: quantitative real time polymerase chain reaction
GEP: gene expression profiling
GEO: Gene Expression Omnibus
SAM: Significant Analysis of Microarray
FDR: false discovery ratio
OS: Overall survival
RFS: relapse-free survival
siRNA: short interfering RNA
*AFAP1-AS1*: actin filament associated protein 1 antisense RNA1
*HOTAIR*: HOX transcript antisense RNA
*GAS5*: growth-arrest-specific transcript 5
