# Supplementary material for: Overexpression long non-coding RNA LINC00673 is associated with poor prognosis and promotes invasion and metastasis in tongue squamous cell carcinoma
Source: Oncotarget. 2016 Dec 25;8(10):16621–32. doi: 10.18632/oncotarget.14200 (PMC5369989; doi:10.18632/oncotarget.14200)
Supplement: Supplementary file 1 [file oncotarget-08-16621-s001.pdf]

Overexpression long non-coding RNA *LINC00673* is associated with poor prognosis and promotes invasion and metastasis in tongue squamous cell carcinoma

SUPPLEMENTARY TABLE

Supplementary Table 1: Clinical information and expression of LINC00673 in TSCC patients.

See Supplementary File 1
